# Extreme Rhabdomyolysis, Acute Renal Failure, and Protracted Ileus in a Case of Legionella Pneumonia

**DOI:** 10.1155/2019/3472627

**Published:** 2019-01-29

**Authors:** C. Laivier, M.-O. Bleuze, P. Hantson, J. Devos

**Affiliations:** ^1^Department of Intensive Care, Centre Hospitalier de Mouscron, 7700 Mouscron, Belgium; ^2^Department of Pneumology, Centre Hospitalier de Mouscron, 7700 Mouscron, Belgium; ^3^Department of Intensive Care, Cliniques St-Luc, Université catholique de Louvain, 1200 Brussels, Belgium

## Abstract

A 53-year-old man developed a* Legionella pneumophila *pneumonia complicated by rhabdomyolysis, acute kidney injury, and protracted ileus. Risk factors were smoking and chronic alcoholism, but the patient had no history of previous abdominal surgery. Hemodialysis was required for a period of 5 weeks with a full renal recovery. Pneumonia required respiratory support but for a limited period of 6 days. The protracted course of the ileus led to explorative laparotomy despite negative computed tomography findings. No cause of mechanical obstruction was found at surgery and common etiologies of intestinal obstruction were excluded. Parenteral nutrition was needed for a total of 4 weeks, before recovery of intestinal motility. This case illustrates the apparent discrepancy between the pulmonary symptoms and the extrapulmonary manifestations that could be seen as a consequence of an exaggerated immune response.

## 1. Introduction

First described in 1977, in the course of a wide community-acquired pneumonia epidemic that occurred during the American Legion's convention, Legionnaires' disease (LD) is an infectious disease caused by an aerobic gram negative bacillus [1].* Legionella pneumophila* is the most frequently encountered species in human pathology, especially serogroup 1 (Lp-1). In over 90% of cases, transmission of LD occurs usually via the respiratory route, after inhalation of contaminated water. Interhuman transmission has never been observed [2]. Several host risk factors were described, including heavy smoking, chronic alcoholism, elderliness, diabetes, end-stage renal or pulmonary disease, and immunodeficiency (corticosteroids, cancer, hemopathy, immunosuppressive therapy, etc.) [3]. LD is a relatively rare infectious disease, with an incidence of 2 cases in Belgium per 100,000 inhabitants in 2016. The mortality rate is still high, ranging from 10% to 70% if the patient is presenting one of aforementioned risk factors [4]. A mild renal impairment is frequent in LD, while extreme rhabdomyolysis leading to acute renal failure and hemodialysis remains uncommon. The association of anuric acute renal failure and persisting ileus has not been previously reported in the setting of* L. pneumophila* pneumonia [5].

## 2. Case Report

A 53-year-old Caucasian man was admitted to the emergency department (ED) with complaints of diarrhea of 3 days' duration, general weakness leading to multiple falls, abdominal pain with lack of appetite, dry cough, and dyspnea on exertion since the last 6 months. His medical past history included chronic alcoholism with 28 units of alcohol per week, active tobacco smoking (22,5 pack-years), sleep apnea syndrome treated with nocturnal continuous positive airway pressure (CPAP) ventilation, high arterial blood pressure controlled by a daily dose of perindopril 5mg and bisoprolol 10 mg, and a morbid obesity with a body mass index (BMI) of 47,3 kg/m^2^. There was no history of abdominal surgery. Vital signs in the ED were arterial blood pressure 106/64 mmHg, sinus tachycardia with heart rate ranging from 104 to 144 bpm, respiratory rate 37/min, temperature 36.2°C, and oxygen peripheral saturation (SpO_2_) 92% at room air. Initial examination revealed crackles at the basis of the right lung and abdominal distension with decreased bowel sounds. There were no clinical signs of muscular injury.

The relevant laboratory investigations were the following: CRP 192 mg/L (< 5), platelet count 115.000 /*μ*L, serum creatinine 4.48 mg/dL, BUN 94 mg/dL, sodium 126 mmol/L, and potassium 3mmol/L. A major rhabdomyolysis was early noted: CK 96,012 IU/L (< 397), troponin-I 103.3 ng/L (< 34.2), AST 818 IU/L (< 37), and LDH 2,960 IU/L (< 214). The CK level further increased to 120,059 IU/L 4 hours after hospital admission. Arterial blood gas analysis was consistent with a compensated metabolic acidosis: pH 7.44, pCO_2_ 21.8 mmHg, pO_2_ 79.7 mmHg, bicarbonate 18.6 mmol/L, base excess -7.2 mmol/L, and lactate level 2 mmol/L (< 1.6). Urine analysis revealed moderate proteinuria (++).

**Figure 1:**
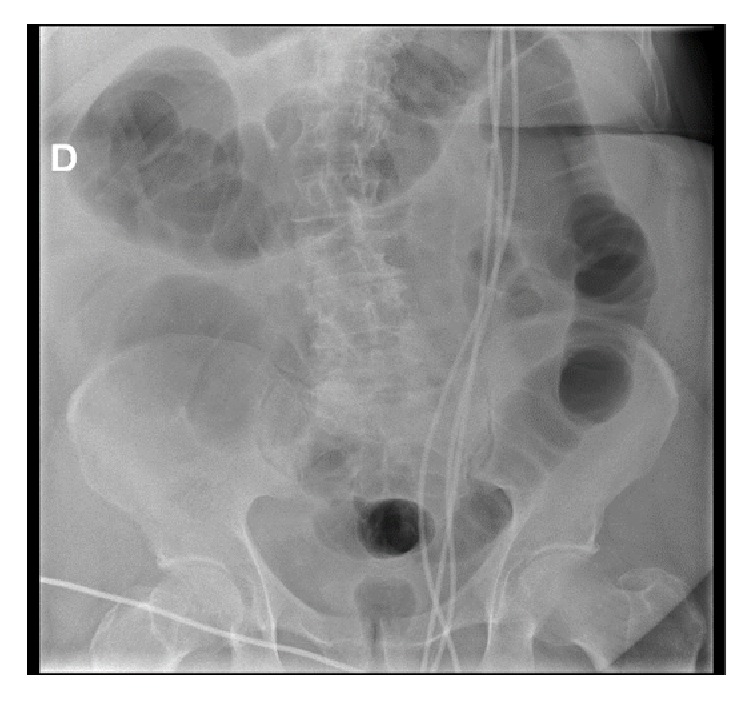
Abdomen X-ray on admission showing diffuse intestinal distension.

The electrocardiogram showed new onset atrial fibrillation (144/min). The diagnosis of left basal pneumonia with a contralateral pleural effusion was made on chest X-ray. Small and large bowel distension was evident from abdomen X-ray with some air fluid levels (Figure 1).

The patient was transferred to the intensive care unit (ICU) for further management. He remained hypotensive (79/43 mmHg), with atrial fibrillation (166/min) and hypoxemia requiring nasal oxygen administration (5L/min). Initial therapy included fluid replacement, vasopressors, and potassium supplementation for persisting hypokalemia (2.9 mmol/L). Cefuroxime and clarithromycin were initially chosen for empirical antimicrobial therapy. No sputum was available for culture and blood cultures remained sterile. Urine* Legionella *testing returned positive for* L. pneumophila* serogroup 1 antigen and clarithromycin was continued alone.

**Figure 2:**
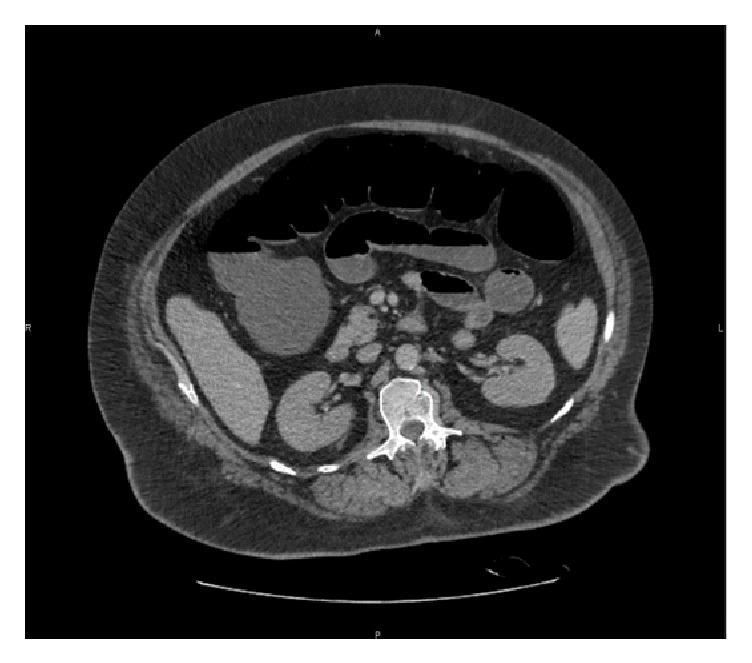
Follow-up abdomen computed tomography (CT) without evidence of mechanical obstruction, inflammatory or ischemic lesions.

Further clinical course was characterized by a progressive weaning of vasopressors and decrease of arterial lactate. Ileus persisted. Contrast enhanced abdominal computed tomography (CT) showed a diffuse distension of the large intestine and terminal ileum and a diffuse enhancement of the intestinal wall without evidence of bowel ischemia or stenosis (Figure 2). Renal failure worsened and the patient became anuric on day 2 with serum creatinine 7.18 mg/dl and BUN 146 mg/dl. Intermittent hemodialysis was then started. The peak level of CK (202,000 IU/L) was reached on day 2.

Mechanical ventilation was ultimately required from hospital day 4 to 10 due to the progression of hypoxemia with hypercapnia; there was also a suspicion of inhalation pneumonia secondary to persisting ileus. During the period of mechanical ventilation, the patient did not receive opioid medications that could have resulted in a decreased intestinal motility.

Despite noncontributive abdominal CT findings, an explorative laparotomy was decided on day 12 that failed to demonstrate any mechanical occlusion.

Two weeks after ICU admission, patient was transferred to the nephrology ward with persisting ileus leading to a full month of parenteral nutrition. Antimicrobial therapy was stopped after 21 days. The patient was discharged home after two months. Hemodialysis was definitely stopped after 5 weeks, and renal function returned to normal values after 6 weeks.

## 3. Discussion

*Legionella pneumophila* infection is an important cause of severe community-acquired pneumonia. The severity of pneumonia may justify ICU admission for respiratory support. In addition, extrapulmonary complications may be encountered following* Legionella* infection. The association of* Legionella* and rhabdomyolysis was first described in 1980 by Posner et al. [6]. Since that time, at least 17 additional cases have been reported in the literature [6–22]. Our patient had one of the highest ever reported CK levels [23]. The exact mechanism leading to muscular injury in case of* Legionella* infection remains unclear. The two main hypotheses are a direct invasion of the muscle by the microorganism or an indirect injury caused by endo- or exotoxin release. The role of ionic disorders may also be discussed as hyponatremia, hypokalemia, and hypophosphatemia have also been related to the occurrence of rhabdomyolysis. Our patient presented with a mild hyponatremia and hypokalemia on admission that were probably insufficient to explain the severity of rhabdomyolysis. From the literature, patients presenting with rhabdomyolysis usually require ICU admission due to the associated renal impairment. At least 13/17 patients needed intermittent hemodialysis, and among them 11 made a complete recovery [22]. The etiology of acute kidney injury (AKI) is likely related to acute tubular necrosis or acute tubule-interstitial nephritis [9]. The presence of* L. pneumophila* was exceptionally demonstrated by immunofluorescence microscopy in a renal biopsy specimen of a patient presenting acute tubulointerstitial nephritis after Legionella infection complicated with rhabdomyolysis and acute renal failure [9]. Other investigations were consistent with tubular precipitation of myoglobin casts [7]. Hypovolemia and hypotension due to gastrointestinal fluid losses may be additional factors for the development of AKI.* Legionella* pneumonia has been exceptionally complicated with Fanconi syndrome [24]. In some cases, renal anomalies can develop before the imaging demonstration of pneumonia.

To our best knowledge, there is no description of the association of rhabdomyolysis-related acute kidney injury treated with hemodialysis and bowel obstruction in the setting of* Legionella* pneumonia. There are isolated observations of segmental enteritis, ascites, or peritonitis following* L. pneumophila* infection [25–30]. Exceptionally,* L. pneumophila* has been demonstrated by direct immunofluorescent microscopic study in inflammatory colitis pieces with hemorrhagic necrosis at different stages [30]. Our patient had suffered from diarrhea during the three days preceding hospital admission. Initial hypotension was likely related to some degree of hypovolemic shock. Prichard et al. described a case of bowel obstruction in a 62-year-old woman with Legionnaires' disease. The patient had a peak CK level at 15,445 IU/L, but with a preserved renal function [5]. The abdomen CT revealed a high-grade partial small bowel obstruction. The patient was managed conservatively. The mechanism of this intestinal complication remained unknown, especially when common factors of intestinal obstruction (previous abdominal surgery, medications, ionic disorders, etc.) could be excluded. The authors hypothesized that the microorganism could activate the immune cascade by the action of lipopolysaccharide, lipid A, on the production of tumor necrosis factor alpha (TNF*α*); the result would be the development of diarrhea and intestinal inflammation [31–34]. A last hypothesis should be a disturbance in gut microcirculation induced by myoglobin casts. Experimental data on rats showed that severe disturbances of the small intestine blood flow may be observed when myoglobin was intravenously infused during a period of hemorrhagic hypotension [35]. Our patient did also not present usual risk factors for intestinal obstruction and gut inflammation is a potential explanation for this uncommon complication. Only one study with multivariate analysis suggested a significant statistical association between diarrhea, rhabdomyolysis, and LD [36]. The association of rhabdomyolysis and renal failure might increase the mortality rate up to 40% [9].

## 4. Conclusion

Among extrapulmonary complications of LD leading to ICU admission, severe rhabdomyolysis and bowel obstruction may be seen as other manifestations of the immune reaction following Legionella infection.
